# PPI Finder: A Mining Tool for Human Protein-Protein Interactions

**DOI:** 10.1371/journal.pone.0004554

**Published:** 2009-02-23

**Authors:** Min He, Yi Wang, Wei Li

**Affiliations:** 1 Key Laboratory of Molecular and Developmental Biology, Institute of Genetics and Developmental Biology, Chinese Academy of Sciences, Chaoyang District, Beijing, China; 2 Graduate School of Chinese Academy of Sciences, Beijing, China; Keio University, Japan

## Abstract

**Background:**

The exponential increase of published biomedical literature prompts the use of text mining tools to manage the information overload automatically. One of the most common applications is to mine protein-protein interactions (PPIs) from PubMed abstracts. Currently, most tools in mining PPIs from literature are using co-occurrence-based approaches or rule-based approaches. Hybrid methods (frame-based approaches) by combining these two methods may have better performance in predicting PPIs. However, the predicted PPIs from these methods are rarely evaluated by known PPI databases and co-occurred terms in Gene Ontology (GO) database.

**Methodology/Principal Findings:**

We here developed a web-based tool, PPI Finder, to mine human PPIs from PubMed abstracts based on their co-occurrences and interaction words, followed by evidences in human PPI databases and shared terms in GO database. Only 28% of the co-occurred pairs in PubMed abstracts appeared in any of the commonly used human PPI databases (HPRD, BioGRID and BIND). On the other hand, of the known PPIs in HPRD, 69% showed co-occurrences in the literature, and 65% shared GO terms.

**Conclusions:**

PPI Finder provides a useful tool for biologists to uncover potential novel PPIs. It is freely accessible at http://liweilab.genetics.ac.cn/tm/.

## Introduction

With the overwhelming amount and exponential increase of biomedical literature, it is almost impossible for biologists to keep abreast of all the updated information in their research fields. Therefore, knowledge-based methods such as text mining techniques to discover hidden and updated knowledge from the unstructured free text are in great need [1]–[3]. One of the most important applications is mining correlations or associations such as protein-protein interactions (PPIs) from the literature [4], [5]. Plenty of PPI text mining approaches have been categorized into two groups, one is statistical calculation of the co-occurrence of genes or proteins, and the other is the computational linguistic method [2], [4].

Statistical methods are based on the hypothesis that if two genes or proteins appeared in the same sentences, paragraphs or articles frequently, there may exist certain kind of biologically meaningful relation between them [2]. Thus, the relations between genes or proteins could be uncovered by calculating their co-occurrence frequencies. In general, the higher the frequencies are, the more likely the interactions are. On the other hand, computational linguistic methods employ natural language processing (NLP) techniques to analyze the semantic meanings of relations (e.g. interaction) between genes or proteins. It first identifies gene or protein names in the sentences. Then it parses the sentences by employing the part-of-speech (POS) tagging. Based on the generated POS tags, a set of predefined protein-protein interaction patterns or rules are applied to extract the protein-protein interaction descriptions [4].

However, the two approaches both have limitations. A drawback of the statistical methods is its inability to tell the exact relations of the genes in co-occurrence. The computational linguistic methods that use one sentence as a processing unit might miss the contextual information [4]. Thus, a hybrid approach by combining the two methods that is termed as a frame-based approach has been developed to have better performance [2].

Biologists may have more interests in the predicted novel PPIs from these text-mining tools. It will be more straightforward to identify potential novel PPIs when the known PPIs are filtered in these algorithms. However, few algorithms have implemented this feature [3]. In this study, we developed a novel algorithm by a frame-based approach for a web-based tool, PPI Finder, which can not only find the related genes of the gene of interest based on their co-occurrence frequencies but also extract the semantic descriptions of interactions from the co-occurring literature by computational linguistic methods. In addition, we map the known interactions from the widely-used PPI databases to filter the known interactions. We also show the shared GO terms from the Gene Ontology database, in order to infer potential PPIs based on their functions in the same process or localization. This dedicated web server is helpful to the users to find both known and potential novel PPIs from literature.

## Methods

### Data Input

#### Homo sapiens-related literature from PubMed:

The current version of PPI Finder only processes related literature in *Homo sapiens* with a volume of 211, 119 PubMed abstracts [6] indexed by gene2pubmed in NCBI Entrez Gene [7] dated from 1950 to 2007, which is controlled by “species = *Homo sapiens*”. The most recent abstracts will be expanded in our system when released annually. We downloaded the XML format files of the literature from PubMed using NCBI Entrez E-Utilities [8].

#### NCBI Entrez Gene Data 2008:

**Figure 1:**
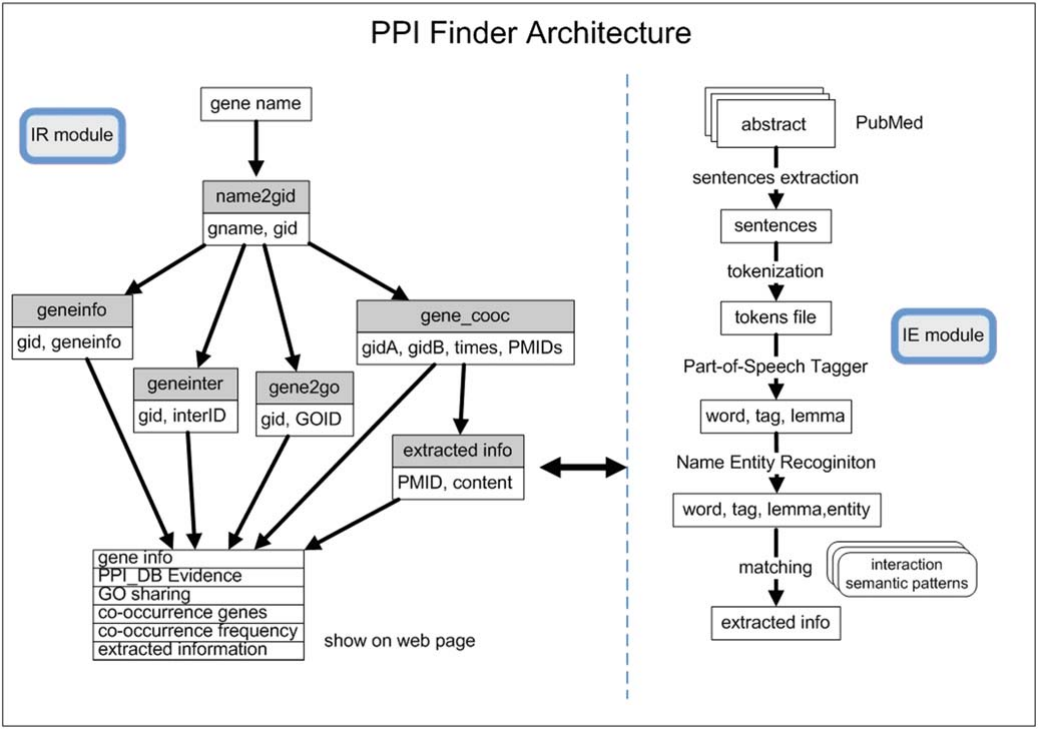
Flowchart of PPI Finder system. PPI Finder system includes two modules: Information Retrieval (IR module) and Information Extraction (IE module). The relationships of the tables and the data structures are described in the text.

We downloaded the NCBI Entrez Gene Data 2008 [7] from ftp://ftp.ncbi.nih.gov/gene/, which is released annually. This includes gene symbols, Gene Ontology (GO) terms [9] and interactions in BIND [10]. The data structure is defined in separate fields and tables (as shown in Fig. 1).

#### Gene2pubmed:

Records the gene-to-literature information including tax_id, gid and PMID, which represents species ID (e.g. *Homo sapiens* = 9606), gene ID assigned in Entrez Gene and PubMed ID assigned in PubMed [6], respectively. We used this table to select the abstracts in *Homo sapiens* and calculate the co-occurrence frequencies between genes (gene_cooc) in different PubMed abstracts.

#### Name2gid:

Records gene's information such as gid, symbol, synonyms, description, other_designations, chromosome, map_location, nomenclature_status, symbol_from_nomenclature_authority, full_name_from_nomenclature_authority. This resolved the redundancy of gene names by using a unique gid in the data structure. In other words, we used this table to implement the search function which can map the user's query with different gene names to a unique gid. This also unifies a gene name and its protein name and records the information of a gene (geneinfo). This feature avoids redundancy in calculating the co-occurrence frequencies when having synonyms of genes.

#### Gene2go:

Records a gene's GO categories (process, component, function) by gid, GO_term and GO ID (GOID) assigned in Gene Ontology [9]. We used this table to provide the GO terms for each gene, allowing users to find the co-occurred GO terms for a given co-occurred gene pair in three GO categories without a hierarchical context.

#### Geneinter:

Records the known interactions controlled by gid (gidA and gidB represents gene IDs of gene A and gene B respectively) and BIND_ID in BIND database [10]. We used this table to provide users with the evidence that the co-occurred genes have known interactions in BIND.

Similarly, we record the interactions in two other well-known human PPI databases: HPRD [11] and BioGRID [12], with the source data downloaded from http://www.hprd.org/download and http://www.thebiogrid.org/downloads.php, respectively.

#### Datasets used in evaluation:

We selected 29 genes listed in the HPSD database [13], for the reason that our laboratory has expertise in studying these genes in order to evaluate the predicted PPIs properly. We randomly selected 100 pairs of genes with known PPIs from the HPRD database [11] to be evaluated for PPI Finder. The expert-confirmed 383 pairs of positive PPIs from the above two datasets, together with 400 pairs of negative PPIs in a matrix of 20 DNA polymerases and 20 ABC transporters, are used to evaluate the sensitivity and specificity of PPI Finder.

### Information Retrieval and Information Extraction

Using the hybrid method of statistical co-occurrence frequencies and computational linguistic analysis, we designed the architecture of the PPI Finder system (see the flowchart in Fig. 1). The PPI Finder system consists of two modules: Information Retrieval (IR module) and Information Extraction (IE module).

#### IR module:

Using the tables of “NCBI Entrez Gene Data 2008”, we constructed the IR module which can map user's query with different gene or protein names to a unique gene ID (gid). The function of mapping different gene or protein names (query) to a unique gid is achieved by the full-text searching features of MySQL 5. Based on the gid, IR module returns the gene's information (geneinfo), co-occurred genes in PubMed abstracts (gene_cooc), interaction evidences (geneinter) from known PPI databases (HPRD, BioGRID and BIND), GO terms sharing (gene2go), and interaction descriptions (extracted info) from co-occurring abstracts (details in IE module).

#### IE module:

The IE module is used to extract interaction descriptions from abstracts of the gene's co-occurring literature. The critical issues we have addressed in this module are described in the following procedures:

We simplified the XML format files of “*Homo sapiens*-related literature from PubMed” by removing the unnecessary tags for more efficient processing in following steps by selection of fields (PubMed ID, Article Title, Authors, Affiliations, Abstract, and Journal).

We employed a natural language processing tool, GENIA Tagger [14], which is specifically tuned for biomedical text processing, to analyze the sentences from abstracts. GENIA Tagger separates the sentences into tokens, tags the part-of-speech of each token, and performs the named entity recognition (recognizing gene and protein names in text).

Based on the processed results of GENIA Tagger, we used the rule-based approach to extract the interaction descriptions. We collected and downloaded the corpus of interaction words or patterns from Huang, et al. [15], Temkin and Gilder, [16], and http://www2.informatik.hu-berlin.de/~hakenber/. These words are commonly used to describe PPIs in biomedical literature. By matching the processed results from GENIA Tagger with the interaction words, we extracted and highlighted the interaction descriptions as well as the gene or protein names.

The extracted results are stored in the database table with the PubMed ID as the index, which are searched to feedback the interaction descriptions in each co-occurring abstract in IR module.

### Implementation

We processed the data and generated the relational database tables using Perl script. All of the database tables were stored in MySQL database system. PHP, JavaScript, Apache HTTP server were used to develop the front end of web application.

### Data Output

**Figure 2:**
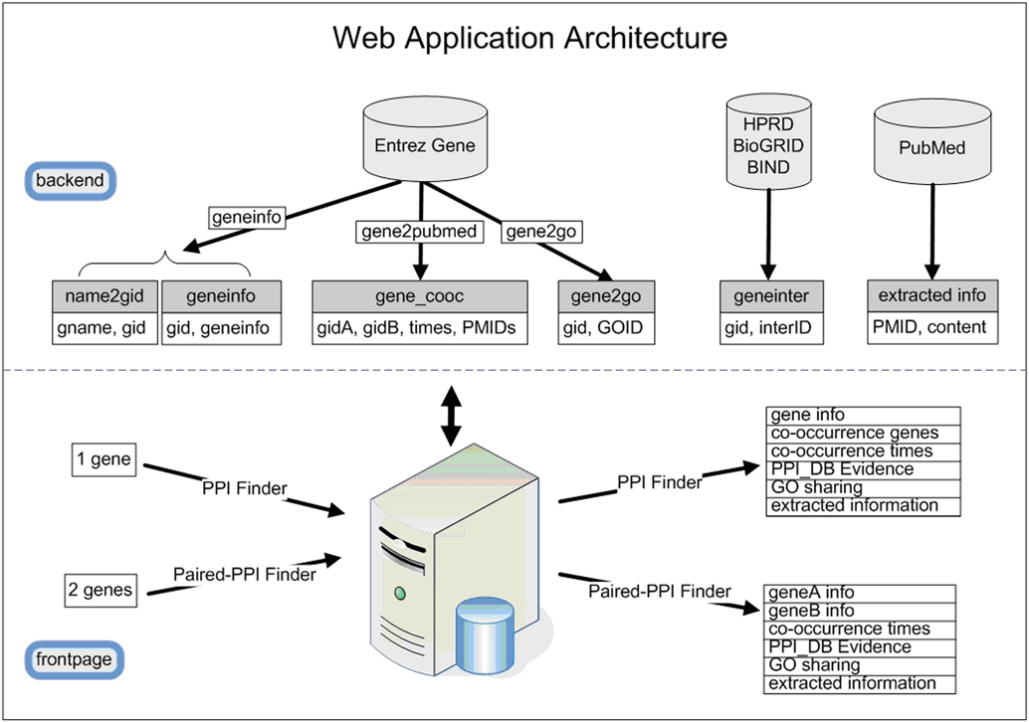
Architecture of the backend and frontpage of PPI Finder. The backend depicts the structure of IR module as shown in figure 1. The frontpage of PPI Finder includes two web applications: PPI Finder (searching one gene at a time) and Paired-PPI Finder (searching two genes at a time). The output format of PPI Finder is summarized.

According to the system architecture (Fig. 1), we developed two web-based applications: PPI Finder and Paired-PPI Finder, which share the same backend database (Fig. 2). They can be freely accessed at http://liweilab.genetics.ac.cn/tm/.

#### PPI Finder Interface:

**Figure 3:**
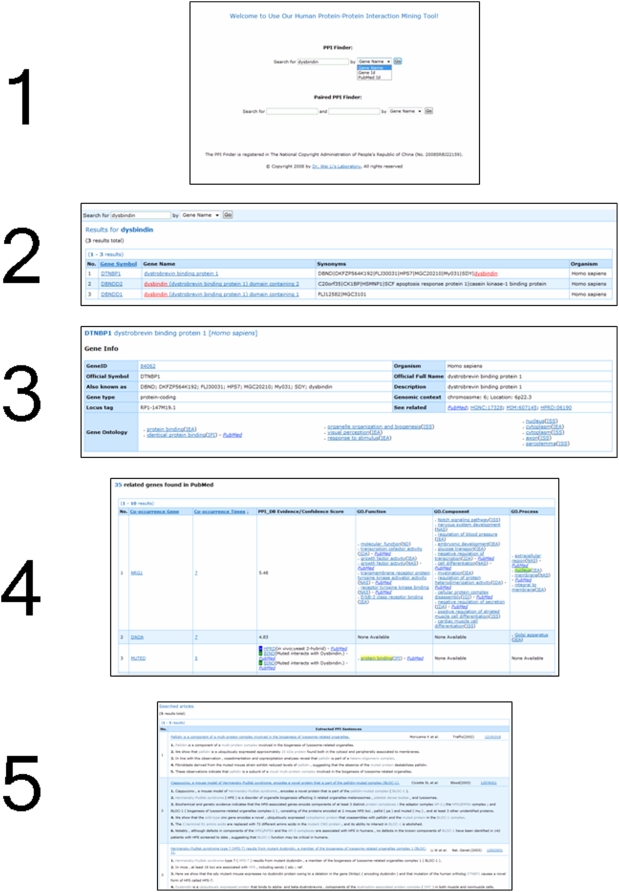
Demonstration of the output results of PPI Finder. Step 1: Our favourite protein name “dysbindin” is searched by selecting “Gene Name”. Step 2: Three results of “dysbindin” are returned. The first row showing “DTNBP1” is the one that unifies the protein name to a unique gene ID. Step 3: By clicking the “DTNBP1” gene, the gene-centred page is shown. The summary of the information of the “DTNBP1” gene is shown on the top. Step 4: The 35 co-occurred genes and their co-occurrence times, PPI database evidences, and gene ontologies are shown at the bottom of the gene-centred page with the co-occurred GO terms highlighted. Step 5: By clicking the hyperlink to “5” in the column of “co-occurrence times” of the fourth co-occurred “SNAPIN” gene, it prompts the co-occurred abstracts and the highlighted words of interaction extraction in the abstracts.

PPI Finder provides three searching options: Gene Name, Gene ID, or PubMed ID (PMID) (Step 1). As for searching by Gene Name, PPI Finder will first return the results of closely matched genes with a query of users' submitted gene or protein name (Step 2). Then, by clicking the prompted gene, users can enter the gene-centred page. The page shows the summary of the gene on the top (Step 3). The co-occurred gene names, co-occurrence times, PPI database evidences, and gene ontologies are shown at the bottom of the gene-centred page with the co-occurred GO terms highlighted (Step 4). By clicking the link to “co-occurrence times”, it prompts the co-occurred abstracts and the highlighted interaction extraction in the abstracts (Step 5). The five steps are demonstrated in figure 3.

Using “search by Gene ID”, users will be directed to the gene-centred page directly. To see the extracted PPI descriptions of a specific article, users can use the “search by PubMed ID” option to view an abstract featured with information extraction processed by NLP. After analyzing the referencing information carefully, users may infer whether a pair of co-occurred genes is a novel PPI candidate.

#### Paired-PPI Finder Interface:

If users are only interested in whether there is an interaction between a specific pair of genes or proteins, Paired-PPI Finder can be used in this situation. Users can input a pair of genes or proteins as queries at one time and will return the related information to infer whether this pair is a known or a novel PPI. Most features are the same as described in PPI Finder.

### Availability and requirements

Project name: PPI Finder.

Project home page: http://liweilab.genetics.ac.cn/tm/

Operating system(s): Platform independent.

Other requirements: Mozilla Firefox or Internet Explorer.

License: The National Copyright Administration of People's Republic of China (No. 2008SRBJ22159).

Any restrictions to use by non-academics: Contact corresponding author.

## Results and Discussion

Except for extracting the interaction words in the abstracts, PPI Finder uses three aspects of referencing information to filter for novel PPIs: co-occurrence times, PPI database evidences (PPI DB Evidence) and GO terms sharing (GO Sharing). We evaluated them by using the following approaches.

### Evaluate the co-occurrence by PPI Finder

We selected 29 genes from the HPSD database [13], searched for them individually by using PPI Finder and returned 944 pairs of co-occurred genes (co-occurrence times ≥1). We counted the PPI DB Evidence as positive when any of the three commonly-used human PPI databases (HPRD, BIND, BioGRID) shows interaction. The evaluation results are shown in Table 1. The low positive rates of PPI DB Evidence (28%) and expert validation (Expert, 33%) of the co-occurred genes suggest the apparent limitations of the statistical methods when based on co-occurrence frequencies only. Similar lower precision rate (∼40%) was referred when applying co-occurrence-based method [17]. Co-occurrence implies that the genes might have a relation but not definitely the PPI relationship. For example, the interaction between a transcription factor and a target gene is often co-occurred in an abstract, but it is not collected in the PPI databases. Similarly, the members of a gene family or the proteins in a same pathway are usually co-occurred in an abstract, but they usually have no interactions. Thus, it is necessary to analyze the text with co-occurrence to see the details processed by computational linguistic method, suggesting the hybrid methods may have better performance. The result that positive rate of Expert (33%) is higher than that of PPI DB Evidence (28%) in the same dataset is probably due to the reason that some of the newly discovered PPIs may have not been expanded into the PPI databases yet.

**Table 1:** Co-occurrence Evaluation.

Co-occurrence	PPI DB Evidence	GO Sharing	Expert
Positive/total	28% (266/944)	53% (504/944)	33% (311/944)
Negative/total	72% (678/944)	47% (440/944)	67% (633/944)

GO terms sharing provide information about the process, component and function of the co-occurred genes. We counted the GO sharing as positive when any of the three categories (function, process, component) is highlighted with the same term. The higher positive rate (53%) of GO sharing suggests lower stringency of GO term matches when making an inference of PPIs.

### Evaluate the PPI database evidences by PPI Finder

We randomly selected 100 pairs of genes with know interactions from HPRD database, searched their co-occurrence and GO terms sharing results using PPI Finder, and submitted them to the experts of the related research fields for evaluation. From Table 2, the positive rate of co-occurrence (69%) suggests about two thirds of the known PPIs are shown in PubMed abstracts. The 31 pairs (31%) that did not show co-occurrences in abstracts are mainly because of the applications of high throughput PPI discovery systems such as yeast two-hybrid, affinity chromatography and Mass Spectrometry. In most cases, the generated data by these techniques are collected in the PPI databases but not recorded in abstracts. Similarly, most of the negative GO sharing (a rate of 35% in Table 2) may be attributable to no assigned GO terms as these PPIs have not been proved by experimental methods and their functions are still unknown.

**Table 2:** PPI Database Evidence Evaluation.

PPI DB Evidence	Co-occurrence	GO Sharing	Expert
Positive/total	69% (69/100)	65% (65/100)	100% (100/100)
Negative/total	31% (31/100)	35% (35/100)	0% (0/100)

Based on this evaluation, we have calculated a confidence score to each item that does not show PPI database evidence in any of those three human PPI databases. A confidence score = (co-occurrence times×0.69)+(GO sharing times×0.65). A higher confidence score may suggest a higher possibility of a putative PPI.

### Evaluate the sensitivity and specificity of PPI Finder

The expert-confirmed 383 positive PPIs and 400 negative PPIs are used to evaluate the sensitivity (Table 3) and specificity (Table 4) of PPI Finder. The co-occurrence showed a sensitivity of 92% and a specificity of 100%, which indicates that our methods of finding co-occurred genes are applicable to information extraction. The PPI database evidences showed a sensitivity of 86% and a specificity of 100%, indicating that most of the PPIs are correctly shown in the databases. The relatively lower sensitivity (66%) and specificity (65%) of GO sharing may be explained by lower stringency of GO term matches as described above, suggesting that it is not a strong indicator of PPI.

**Table 3:** Sensitivity Evaluation.

Expert Positive	Co-occurrence	PPI DB Evidence	GO Sharing
True positive/total	352/383	329/383	252/383
False negative/total	31/383	54/383	131/383
Sensitivity	92% (352/383)	86% (329/383)	66% (252/383)

**Table 4:** Specificity Evaluation.

Expert Negative	co-occurrence	PPI DB Evidence	GO Sharing
False positive/total	0/400	0/400	140/400
True negtive/total	400/400	400/400	260/400
Specificity	100%	100%	65%

Taken together, the evaluation results reveal that PPI Finder's applications in PPI database evidence, GO sharing and extracted descriptions from co-occurred abstracts provide multiple tiers to assist biologists to infer the novel PPIs hidden in literature.

Comparing with the existing text mining systems (such as iHOP, PubGene, PIE) for discovering PPIs [3], [18], PPI Finder has several unique features by adopting the hybrid methods with statistical and computational linguistic theories. These include (1) PPI Finder filters the known PPIs; (2) PPI Finder provides the information of shared GO terms; (3) PPI Finder is a search engine for interaction of any given pair of genes. Although we use the PubMed abstracts in this study, there is no limit to the length of an article when using PPI Finder. The full-length article may provide more information for text-mining, but the current freely accessed full-length papers are limited. One limitation of PPI Finder is that the data we used are offline. We need to update the backend databases whenever there are new data released from NCBI Entrez Gene or PubMed. Future versions of PPI Finder will be applied to other species such as mouse and fly.

In summary, we developed PPI Finder which uses a hybrid text mining approach combining statistical and computational linguistic methods. By using PPI Finder, biologists can search their genes of interest and may uncover some novel PPIs from published biomedical literature.
